# Synthetic Methodologies and Therapeutic Potential of Indole-3-Carbinol (I3C) and Its Derivatives

**DOI:** 10.3390/ph16020240

**Published:** 2023-02-05

**Authors:** Federica Centofanti, Alessandro Buono, Michele Verboni, Carlo Tomino, Simone Lucarini, Andrea Duranti, Pier Paolo Pandolfi, Giuseppe Novelli

**Affiliations:** 1 Department of Biomedicine and Prevention, Tor Vergata University of Rome, 00133 Rome, Italy; 2 Department of Biomolecular Sciences, University of Urbino Carlo Bo, 61029 Urbino, Italy; 3 Scientific Direction—IRCCS San Raffaele Rome, 00166 Rome, Italy; 4 William N. Pennington Cancer Institute, Renown Health, Nevada System of Higher Education, Reno, NV 89502, USA; 5 Department of Pharmacology, School of Medicine, University of Nevada, Reno, NV 89557, USA

**Keywords:** Indole-3-carbinol, phytochemical, natural products, in vitro assays, in vivo tests, anticancer activity, antiviral activity

## Abstract

Indole-3-carbinol (I3C) is a natural product contained in vegetables belonging to the *Brassicaceae* family and has been studied in recent decades for its biological and pharmacological properties. Herein, we will analyze: (1) the biosynthetic processes and synthetic procedures through which I3C and its main derivatives have been obtained; (2) the characteristics that lead to believe that both I3C and its derivatives are responsible for several important activities—in particular, antitumor and antiviral, through insights concerning in vitro assays and in vivo tests; (3) the mechanisms of action of the most important compounds considered; (4) the potential social impact that the enhancement of the discussed molecules can have in the prevention and treatment of the pathologies’ examined field—first of all, those related to respiratory tract disorders and cancer.

## 1. Introduction

An unbalanced diet with low amounts of fruits, vegetables, whole grains, seeds, and nuts, and an exaggerated consumption of ultra-processed foods is the leading contributor to chronic disease risks. Instead, there is a clear correlation that high consumption of fruits and vegetables plays a central role in the prevention of non-communicable diseases [1].

**Figure 1:**
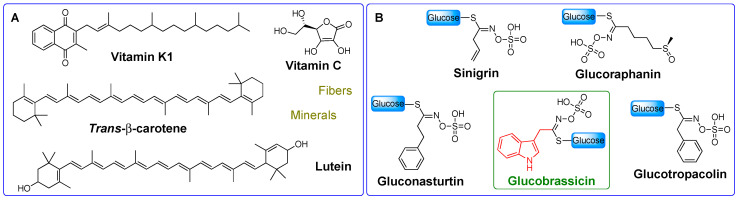
(**A**) Representative high nutritional value compounds contained in cruciferous vegetables. (**B**) The main bioactive glucosinolates found in cruciferous vegetables.

Cruciferous vegetables, which belong to the *Brassicaceae* family, such as broccoli, broccoli sprouts, brussels sprouts, cabbage, cauliflower, kale, and green cabbage [2] are very nutritious foods in the diet, as evidenced by many clinical and epidemiological studies [3] and contain countless compounds with high nutritional value and proven beneficial effects, including fiber, vitamins, minerals, *β*-carotene, lutein, and phytochemicals [1] (Figure 1).

A diet poor in phytochemical content could lead to risks, such as in the cardiovascular field, because its deficit favors inflammation and oxidative stress (reducing nitrogen monoxide levels) and, consequently, atherosclerosis, endothelial dysfunction, and vascular inflammation [4]. Some studies have reported the anticarcinogenic activity of several metabolites found in cruciferous vegetables [5], such as those of the gastrointestinal tract [6].

The main bioactive compounds found in cruciferous are glucosinolates, which are sulphur-rich, anionic, and water-soluble secondary metabolites, such as glucobrassicin and derivatives (Figure 1). These molecules, based on the different side chain of the amino acid precursor, are divided into three groups: aliphatic (methionine, isoleucine, leucine, or valine), aromatic (phenylalanine and tyrosine), and indole (tryptophan). In this context, various processes lead to the biosynthesis of more than 200 glucosinolates, but only some of these are biologically active and could potentially show beneficial effects on humans [2].

**Figure 2:**
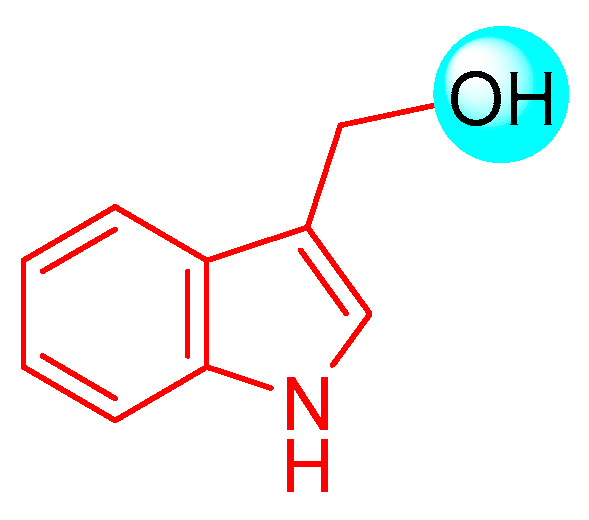
Chemical structure of I3C.

A bioactive compound belonging to the *Brassicaceae* family that has been extensively studied is indole-3-carbinol (I3C, Figure 2) [7,8]. The focus of this review is to analyze both the synthetic procedures through which I3C and its main derivatives were obtained, and the characteristics deriving from the results obtained through bioassays and pharmacological tests. With regard to I3C derivatives, the purpose of this review is to evaluate the natural dimeric, trimeric, or tetrameric derivatives, which help to understand further direct information about the structural requirements for the activity. Monocyclic analogues of I3C will, therefore, not be considered.

## 2. I3C Natural and Exogenous Synthesis, In Vitro and In Vivo Activity, and Mechanism of Action

I3C is an important phytochemical contained in cruciferous vegetables and is able to exert various activities among which are cardioprotective, antioxidant, anti-inflammatory, antiangiogenesis, and antimicrobial activities, the promotion of tumor cell apoptosis [4,9] and, more recently, an important inhibition of Severe Acute Respiratory Syndrome Coronavirus 2 (SARS)-CoV-2 viral egression, including the Omicron variant [10,11].

I3C is often present in dietary supplements, since its uptake has been related to a lower risk towards several diseases, such as those related to the pathology previously considered. Indeed, it is the subject of on-going biochemical and pharmacological research for its possible beneficial effects.

I3C exhibits antitumor activity in different cancer cell lines. The pioneering works that highlighted the anticancer properties of I3C date back to 1975 from Wattenberg et al., which described for the first time how I3C and its derivatives as molecules are endowed with a protective effect against chemical carcinogens [12,13]. When administered in the diet, it significantly decreased the incidence of spontaneous and chemically induced mammary tumors in mice [14]. Furthermore, a reduction of pulmonary metastases was found in mice injected with mammary tumor cells and fed with cabbage [15]. However, the development of I3C in the prevention of breast cancer is limited due to its instability in an acidic environment, which would force the administration of a relatively high concentration to block the growth of the tumor with the risk of reaching quantities that do not fit in the therapeutic window [16].

### 2.1. I3C Biosynthesis

**Figure 3:**
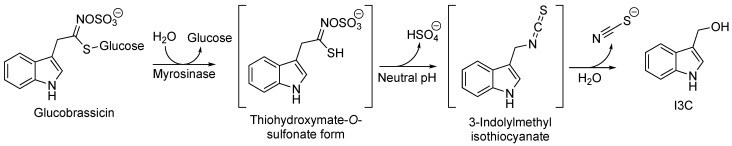
Biosynthesis of I3C starting from glucobrassicin.

With regard to the environment of plants, the role of glucosinolates is to protect them. Upon damage to plant tissues, they undergo enzymatic hydrolysis and are transformed into bioactive molecules toxic to herbivores and pathogens. When plant tissues are damaged, the β-thioglucosidase [17] enzyme myrosinase [18] can hydrolyze the thioglucosidic bond in the structure of the glucosinolates [2]. In particular, I3C is obtained from the degradation of the glucosinate glucobrassicin. This process is carried out through the formation of unstable intermediates thiohydroximate-*O*-sulfonate and 3-indolylmethyl isothiocyanate, which is finally converted into the desired compound I3C (Figure 3).

### 2.2. I3C Synthetic Methodologies

Among the numerous synthetic methodologies followed to obtain I3C, those with the best characteristics to be reproduced on a very large scale (the first illustrated) and the one with very high yield were selected.

**Scheme 1:**
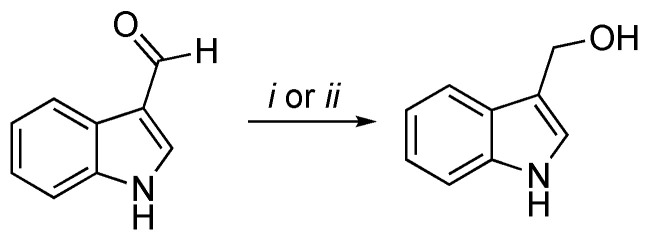
Reagents and conditions: (*i*) NaBH_4_, MeOH, 0 °C, 30′; (*ii*) NaBH_4_, EtOH/THF, rt, 3 h.

A classical method to obtain I3C requires highly basic conditions such as the reduction of the corresponding aldehyde with sodium boron hydride (NaBH_4_) [19,20], as follows in Scheme 1.

**Scheme 2:**
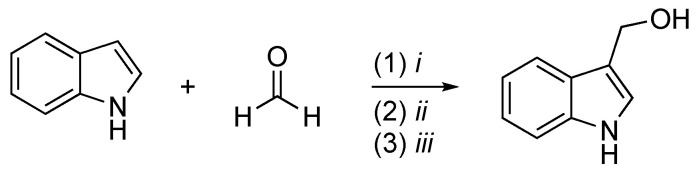
Reagents and conditions: (*i*) TMSOTf, *i*-Pr_2_NEt, Et_2_O, N_2_, −78 °C, 1 h; (*ii*) pyridine; (*iii*) TBAF, THF, rt, 5′.

The second scheme describes the method to obtain I3C from indole proposed by Downey et al. [21] (Scheme 2). Indoles undergo Friedel–Crafts addition to aldehydes in the presence of trimethylsilyl trifluoromethanesulfonate (TMSOTf) and a trialkylamine to form 3-(1-syloxyalkyl)indoles. The reaction is quenched by the addition of pyridine followed by deprotection under basic conditions with tetrabutylammonium fluoride (TBAF) and provides the free desired product. This method prevents the formation of bisindolyl methanes, a thermodynamically favored process typically observed when indoles react with aldehydes under acidic conditions.

As mentioned before, I3C is a well-researched and interesting compound due to its extensive biological properties and may activate multiple antiproliferative cascades [22].

For an extended period, I3C has been widely explored for potential roles in different cellular mechanisms, including the suppression of cell cycle progression, blocking of cancer cell migration, the promotion of apoptosis, and the inhibition of tumor growth [23,24,25]. Although it interacts with different pathways, it has been proposed that I3C and its synthetic derivatives may influence human cells by directly inhibiting them with specific enzymatic target proteins. Specifically, they are potent natural inhibitors of homologous to E6AP carboxyl-terminus (HECT) family members of E3 Ubiquitin-Ligase, emphasizing the potential importance of I3C in developing highly potent and stable anti-cancer molecules, and not only [26]. I3C and its derivatives are also able to inhibit neuronal precursor cell-expressed developmentally downregulated 4 (NEDD4) and WW domain-containing ubiquitin E3 ligase 1 (WWP1) ubiquitination enzymatic activity through the interaction with their catalytic subunit [26,27,28,29].

Since I3C affects many cellular mechanisms, it is not surprising to find proposed applications in the treatment or prevention of different cancer types (e.g., breast, ovarian, prostate, lung, liver, and colon) and in other diseases [30,31,32,33,34,35,36,37,38]. To date, it has been tested in many clinical trials for the prevention and treatment of obesity, chronic inflammation, lupus erythematosus (SLE), and breast, colon, and prostate cancer [39]. Details about all of the applications of the potential of I3C and its derivatives in different pathologies are reported below.

### 2.3. I3C Antitumor Activity: In Vitro, In Vivo, and Clinical Studies

Cancer is one of the leading causes of disease and death worldwide and is defined as uncontrollable and abnormal cell growth [40]. In breast cancer, gland cells grow abnormally and uncontrolled, resulting in the development of a malignant and metastatic phenotype [41]. It is divided into four molecular subtypes based on the expression of the estrogen receptor (ER), progesterone receptor (PgR), and the epidermal growth factor receptor 2 (HER2): two are characterized by the presence of hormone receptors, which provide hormonal therapeutic treatment; then, there is the HER2 subtype, which constitutes about 20% of all cases and is a biologically treatable tumor with some monoclonal antibodies specific to the substance used; finally, there is the triple-negative tumor (it has no receptors for either hormones or HER2), which is sometimes more aggressive and is subject to studies aimed at specific therapies [42].

Although conventional treatments are clinically effective against breast cancer, they present severe side effects associated with the development of resistance and high toxicity in healthy cells leading to a poor prognosis [40]. Therefore, the discovery of new treatments and in particular, the use of natural compounds is one of the primary goals in the field of breast cancer biology, due to therapeutic potential with minimum side effects compared to the traditional methods such as chemotherapy and radiotherapy [43].

In this context, different studies have been conducted on different phytochemical compounds that have brought to light significant antitumor effects, preventing malignant neoformations, inhibiting the metastatic process, and also optimizing first-line anticancer therapies [41]. For many years, several studies have highlighted the potential benefits of the phytochemical compound I3C (and its derivatives; in particular, DIM) as promising antitumoral options with negligible toxicity [23,44,45,46], due to the fact that many of these antiproliferative responses are selectively controlled by I3C-activated pathways. Now, we will illustrate in molecular detail how I3C and its derivatives exert their antitumor activity in the human cancer cell.

Regardless of the type of tumor, I3C triggers DNA repair, cell-cycle arrest, apoptosis, disruption of cell migration, and modulates hormone receptor signaling [47,48,49]. The first identified I3C target protein was serine protease elastase [50]. In human breast cancer, it has been observed that inhibition of elastase activity by I3C prevents CD40 cleavage, resulting in the disruption of the NFkB-dependent cell cycle and proliferation [51,52].

Moreover, I3C and its derivatives also play a fundamental role in DNA damage repair processes by influencing the expression of tumor suppressor genes such as BRCA1 and BRCA2 and DNA repair proteins (i.e., RAD51, which regulates responses in the case of DNA damage) [53,54]. In all estrogen-dependent tumors (including breast cancer), I3C inhibits cyclin-dependent kinase (CDK) expression, resulting in the stopping of the cell cycle in the G1 phase. In particular, I3C has also been reported to induce apoptosis in the PC-3 human prostate cancer cell line by inhibiting the activation of serine/threonine kinase Akt [55,56]. In addition, I3C has the ability to counteract the metastatic process, tumor angiogenesis, and migration of cancer cells, inhibiting CDK6 with the consequent blockage of cell growth [57]. These observations suggest that I3C may mediate its anti-proliferative effects by directly interacting with other classes of target proteins with enzymatic activities.

More recently, through the identification of genes involved in tumorigenesis and cancer susceptibility, it was found that members of the HECT family of E3 ubiquitin ligases were often over-expressed in human cancers. They display oncogenic properties through the ubiquitin-dependent regulation of several protein substrates such as phosphatase and tensin (PTEN) homolog, a negative regulator of phosphatidylinositol-3,4,5-trisphosphate and the Akt/PKB signaling pathway [29]. Specifically, two members of a subgroup of HECT-E3 ligases, known as C2-WW-HECT (NEDD4-like), have been identified to be most involved in cancer: NEDD4 and WWP1. They are characterized by WW domains that act on protein–protein interactions by recognizing Pro-rich and Ser/Thr-Pro phosphorylated patterns. These domains provide scaffolding to recruit protein substrates and regulators [26,27,28].

Quirit et al. have demonstrated through in vitro assay that I3C directly inhibits NEDD4 ubiquitination activity. Moreover, protein thermal shift assays, in combination with the in silico binding simulations and crystallographic structure of NEDD4, showed the binding involvement to the purified catalytic HECT domain of NEDD4 [26]. Likewise, due to its structural similarity to NEDD4, WWP1 was also demonstrated to be inhibited by I3C. It is interesting to note that the MST (microscale thermophoresis) binding assay found that I3C binds to the WWP1 HECT domain with a dissociation constant much higher than that related to NEDD4 [27].

In addition to directly influencing specific molecules, I3C (and DIM) also seem to be able to regulate gene expression by modulating DNA methyltransferases, histone deacetylases, miRNAs, lncRNAs, and some transcription factors such as the aryl hydrocarbon receptor (AhR), ER, and NF-kB [58,59,60]. In fact, we know, for example, that DNA methylation involves the silencing of tumor suppressor genes, an important mechanism for developing target molecules for chemoprevention, and able to modulate the methylation of these genes. In this regard, it has been seen that the inhibition of DNMT (DNA methyl transferase) by DIM has shown a lower expression of oncogenes and an increase in tumor suppressor genes [61]. This mechanism has emerged from some experiments on mouse models and on human prostate cancer cells, on healthy prostate cells (PrEC), as well as on androgen-dependent (LnCAP) or androgen-independent (PC3) prostate cancer [62].

These results suggest that I3C could provide a starting point to develop highly potent anticancer compounds based on target-protein interactions to have a more potent antiproliferative response in human breast cancer cells (and also for other types of cancer) compared to other inhibitors [52].

In recent years, the demand for natural products with prophylactic action against a series of diseases and their adverse reactions is increasing. According to some studies, I3C exerts a potential prophylactic action either directly or through its metabolites [63].

Recently, Lee et al. identified, in either Myc-driven or PTEN heterozygous mice, the reactivation of PTEN due to a pharmacological inactivation of WWP1 by I3C, leading to the potent suppression of tumorigenesis driven by the PI3K-Akt pathway [27]. Moreover, PTEN deletion by CRISPR-Cas9 in Hi-Myc tumor organoids conferred partial resistance to I3C, supporting that I3C exerts its function in a PTEN-dependent manner. In addition, an I3C pharmacokinetic analysis in male C57BL/6 mice after intraperitoneal administration at 20 mg/kg every day or every other day was carried out. These results support again that I3C targets WWP1 E3 ligase, triggering reactivation of the PTEN tumor suppressive function, and promoting its plasma membrane recruitment, and suppression of MYC-driven tumorigenesis in vivo. These findings highlight the use of I3C as a potential therapeutic strategy to reactivate PTEN (through WWP1 inhibition) and to treat all of those patients with multiple tumors or other diseases associated with PTEN germline mutations and for cancer prevention, through the targeting of WWP1-PTEN axis pharmacologically [27].

There are also several studies carried out on mouse models to test the effectiveness of I3C and its derivatives. Studies on immunodeficient mice, such as the naked mouse or the non-obese diabetic (NOD) mouse, in which human cancerous cell lines (xenotransplantations) are implanted and follow the dietary administration of I3C (or DIM), have observed the inhibition of tumor cell proliferation over time [64,65,66]. Again, following the implantation of human cancer cell lines in SCID (severe combined immunodeficiency) mice (NOD. CB17-Prkdcscid/SzJ), the efficacy of I3C in comparison to that of DIM from food in inhibiting tumor growth has been analyzed: both tumor size and doubling time of human T-ALL CCRF-CEM cell xenografts in these mice were significantly affected by 100 ppm of dietary DIM, while they were sensitive to 500 and 2000 ppm of I3C from the diet [64]. The chronic administration of I3C in rodents has led to the development of hepatocarcinogenesis [67,68], while in the infantile model, it led to a high decrease of liver tumors induced by diethylnitrosamine [69]. Some studies on colon cancer have brought to light activity of chemoprevention of I3C and DIM according to an AhR-dependent modality, also susceptible to the microbial production of indole AhR ligands starting from the metabolism of dietary tryptophan [70,71]. For the analysis of breast cancer in rats induced by DMBA (7,12-dimethylbenzanthracene) or MNU (*N*-methylnitrosourea), a direct-acting carcinogen, I3C, unlike DIM, has been shown to be effective in chemoprevention [72]. All of this evidence justifies the ability to attribute I3C chemopreventive activity to DIM in each study performed. Protection against TBD-dependent in female, but not male, offspring originating from mothers fed I3C during gestation required the expression of ERβ [73]. These results would be in line with the chemoprevention of I3C and ER-dependent DIM in breast cancer [23,74,75,76,77,78,79,80,81].

Preclinical studies of I3C as chemopreventive agents have yielded excellent results [24,82,83]. For example, in addition to liver, breast, and colon cancer, dietary I3C leads to a decrease in lung carcinogenesis caused by the specific nitrosamine contained in tobacco, 4-(methylnitrosamino)-1-(3-pyridyl)-1-butanone, PAH (pulmonary arterial hypertension), and BAP (broader autism phenotype) [84].

Since numerous in vitro and in vivo studies have shown its powerful chemopreventive action in different cancers, I3C has also been the subject of some promising clinical studies concerning cancer therapy. In a double-blind placebo-controlled study, the ability of I3C to prevent the onset of breast cancer was evaluated [85]. The study involved 60 women aged 22 to 74 years who received doses of 50 mg or 400 mg of I3C and in no case did adverse effects occur except in patients with a history of elevated aminotransferase levels. The study showed that I3C exerts a preventive effect on breast cancer and all forms of estrogen-dependent cancer [85]. It is important to consider that breast cancer in women has been placed in the foreground following the proven activity of I3C (and DIM) to act on CYP1-dependent estrogen metabolism. With regard to the metabolites of estradiol (E2), 2-hydroxyestrogen has a lower pharmacological activity, in contrast to 16-α-hydroxyestrogen, which maintains estrogenic activity. The ratio (2-hydroxyestrogen)/(16-α-hydroxyestrogen) to date is considered a biomarker for the identification of the risk of an estrogen-dependent tumor and for cervical intraepithelial neoplasia (CIN), such that clinical studies carried out with I3C (and DIM) have determined an increase in the ratio between the two estrogens, and therefore, the prevalence of the non-estrogenic metabolite, and, in the specific case of CIN, an improvement in the pathology.

I3C (and DIM) also affect the action of flavin-containing monooxygenases, i.e., a superfamily of enzymes contained in the endoplasmic reticulum of tissues such as the liver, lung, intestine, and kidney, which catalyze redox reactions in which NADPH is identified as a reducing agent and, at the same time, an O_2_ atom is added in a substrate while the other is used for the formation of H_2_O [86]. Humans express five FMOs (FMO 1–5) but in mammals, excluding primates, FMO1 is the main form present in the liver capable of metabolizing a wide range of xenobiotics [87]. Specifically, dietary I3C (and DIM) inhibits FMO1 only in rats and FMO3 in humans [88,89,90,91,92]. The ratio of FMO/CYP-mediated metabolism of DMA was reduced in a dose-dependent manner following the intake of dietary I3C (and DIMs). When considering (*S*)-nicotine, the rate of metabolism operated by CYP did not change with diet, while *N*-oxygenation was significantly inhibited, and no forms of *N*-oxide were detected in liver microsomes of rats fed 2500 ppm I3C (or 1000 ppm DIM). Tamoxifen, an estrogen receptor antagonist ER used in chemoprevention or treatment of ER-dependent breast cancer, allows limited use from ovarian toxicity due to the hydroxylation reaction at position 4 [93]. Again, the *N*-oxidized form is the eliminable form and the inhibition of *N*-oxide metabolism is likely to increase the toxicity of both (*S*)-nicotine and tamoxifen [94].

However, the actual clinical and preclinical I3C concentrations as well as the consumption of large quantities are still unclear; a recent paper published by Centofanti et al. evaluated potential adverse in vivo toxicity effects through two different routes of administration [intraperitoneally (i.p.) and intragastrically (i.g.)] in both male and female mice, because in females, the effect of I3C may be subject to hormonal changes. This analysis showed a different tolerability dose in males and females to be considered for any future clinical trials in which I3C will be used. Overall, below 550 mg/kg for i.g. and 250 mg/kg for i.p. values, I3C induces neither death nor abnormal toxic symptoms, as well as no histopathological lesions [11].

It is always important to remember that the doses used in preclinical and clinical studies are significantly higher than the doses that can be derived from food sources; for this reason, supplementation is essential to achieve the same health effects attributed to I3C (and DIM).

Finally, it is also important to highlight some controversies that emerged from different studies on the cellular effect of high I3C concentration. Rather than I3C, this effect should be attributed to its DIM derivative(s), because I3C rarely, if ever, is detected in the blood after oral ingestion [95]. Moreover, it has been proven that in humans, a significant portion of DIM is actually present in the form of its metabolites, having an unknown pharmacological activity [96].

Although I3C and DIM have been subjected to clinical trials in humans, essentially to investigate their efficacy against breast and prostate cancer [59,95,97,98,99,100,101,102], different epidemiological studies have been carried out on the population that have brought to light a negative relationship between the intake of cruciferous vegetables and some cancers [83,103,104,105,106].

Notably, except for one case, these clinical studies consider the therapeutic potential of DIM in cancer and not the chemopreventive activity, which in fact requires double-blind, placebo-controlled studies in disease-free subjects to confirm its potency and efficacy as a supplement.

### 2.4. I3C Anti-Inflammatory Activity: In Vitro, In Vivo, and Clinical Trials

I3C exerts a potent anti-inflammatory activity in ischemia-reperfusion processes, a situation characterized by an intense interaction between leukocytes and endothelial cells. The administration of I3C, in fact, suppresses the expression of E-selectin, a glycoprotein involved in the interaction between leukocytes and endothelium and in the consequent transmigration of leukocytes, and, thus, decreases the transcriptional activity of the nuclear factor NF-kB in B cells. In accordance with this observation, treatment with I3C reduces the number of leukocytes adhering to the injured site, with remarkable anti-inflammatory effects [107].

In several circumstances, I3C has established itself as an anti-inflammatory agent, in particular in ischemia-reperfusion lesions, an inflammatory situation and tissue damage resulting from the restoration of blood flow in a certain compartment previously characterized by ischemia or hypoxia. During this process, there is a remarkable interaction between circulating leukocytes and endothelial cells residing at the site of injury. Thus, I3C interferes by inhibiting the expression and production of glycoproteins having the fundamental role of mediators of the interaction between the two cell types (i.e., E-selectin) and consequently blocks the subsequent transmigration of leukocytes by reducing the transcriptional activity of the nuclear factor enhancer of the kappa light chain of activated B lymphocytes (NF-kB) [107].

Based on these studies, it is clear that I3C may be beneficial for the treatment of inflammatory diseases.

As we know, inflammation is the first defensive response induced by the body’s immune system against foreign molecules and exogenous agents (such as pathogens), although it can result in different inflammatory patterns due to the establishment of the acute phase response or chronic inflammation.

Systemic lupus erythematosus (SLE) is an autoimmune disease of a chronic character, which, gradually, can cause inflammation of the joints, skin, blood cells, kidneys, lungs, fundamental elements of the nervous system, heart, and other organs. The pathogenesis of SLE is still being studied because the onset of the disease depends on multiple factors [108]. Several studies have shown that SLE is related to an abnormal state of the immune system. In SLE patients, the distribution of T cell types is abnormal, and in particular, is characterized by a decrease in the ratio between Th cells (helper) and Ts (suppressor), an imbalance of the Th1/Th2 ratio and a decrease in Treg cells [108]. They also have several abnormalities affecting B cells. In general, B cells have a higher memory, are more resistant to immunosuppressive agents, and are activated more easily. Since, as already considered, B cells and T cells communicate with each other, abnormal activation of B cells also causes aberrations in the activity of T cells and imbalances in the release of cytokines. SLE is also characterized to aberrations in the functionality of macrophages, with consequence dysregulation of cytokine production. Defective macrophages are considered to be the main propagation factor of SLE [109].

Currently, SLE is a disease which is impossible to cure, although there are several treatments to alleviate its symptoms and control its complications [108]. Nowadays, SLE is treated with four classes of drugs: antimalarials (such as chloroquine, hydroxychloroquine, and dihydroxyartemisinin), glucocorticoids, immunosuppressants (including cyclophosphamide, methotrexate, and leflunomide) and biological agents (abatacept and cryptotasinone). However, I3C could help in disease management.

Different mechanisms have been proposed concerning the control of macrophage functions, but the most accredited pathways are those mediated by the PPAR-γ receptor (peroxisome proliferation-activated receptor gamma), histone deacetylase (HDAC), and AhR. AhR is a cytoplasmic receptor and transcription factor that plays an active role in detoxification from environmental pollutants.

I3C is an AhR receptor agonist with anti-inflammatory properties; therefore, it is a good candidate for therapy. In addition, it is known that I3C improves estrogen metabolism by diverting it towards the formation of less toxic derivatives (2-hydroxyhexatrone vs 16-α-hydroxyhexatrone), and this anti-estrogenic activity can also counteract SLE, which is influenced by estrogen metabolism. I3C also possesses regulatory properties of inflammation by acting on the differentiation of T cells and B cells [110].

It has been demonstrated in (NZB × NZW) F1 mice experiments that I3C is able to block SLE disease progression and prolonged survival. Specifically, in treated animals, a transient blockade in B-cell maturation occurs with increased immature B cells, decreased mature B cells, and a significant reduction of certain autoantibodies. Subsequently, a delay in T cell maturation occurred in the treated group, manifested by significantly increased naive T cells, decreased mature and memory T cells, and decreased CD4:CD8 T cell ratios. Moreover, T cells from the I3C cohort, stimulated in vitro with various mitogens, exhibited enhanced responsiveness. This study suggests immunological mechanisms by which I3C ameliorates SLE in mice and provides a rationale for its use as an adjunctive therapy for human lupus [111].

In another study still performed on female (NZB × NZW) F1 mice, it was observed that I3C, given as a dietary supplement, dramatically increases the lifespan of lupus-prone mice and is more effective when initiated early, before the onset of disease, or later when the disease develops. The results confirm that I3C can be beneficial to people at risk of SLE as well as those in the early stages of the disease. As a result, I3C can reduce the dose of immunosuppressive drugs needed to treat SLE, reduce toxic side effects, and be helpful in preventing recurrence. This study adds I3C to the list of nutrients that may have beneficial impacts on SLE results [112].

All of these observations led to the study of I3C as a potential drug for SLE for clinical studies.

One of the first clinical studies evaluated the effect of I3C administration on 80 female patients with a definitive or probable diagnosis of SLE, up to 40 years of age during menstruation or plasma follicle-stimulating hormone (FSH) levels below 30 mlU/mL [113]. Patients took doses of 125 mg of I3C orally 3 times/day for a period of 3 months. They were followed and systematically monitored at weeks 0, 1, 4, 8, 12, and 24 after the start of the study to determine the SLE disease activity index (SLEDAI) and any side effects. The study showed that patients with SLE respond after administration of I3C, with a significant change in the ratio 2-hydroxyhexatron/16-α-hydroxyhexatrone to increased estrogen 2-hydroxylation, a situation that encourages the use of I3C in SLE therapy. In particular, this study confirmed that I3C has an effect on estrogen metabolism in women with SLE that can improve their health. The compound is well tolerated but studies on the effective action against the disease need to be investigated [113].

A recent study evaluated the effect of I3C on the polarization of leukocytic macrophages mediated by activation of the AhR in SLE patients [110].

The study sample consisted of 15 patients diagnosed with SLE, while the control consisted of 10 healthy subjects of the same age group; all subjects were female. Isolated macrophages from these patients were treated with I3C at 100 μM and evaluated phagocytosis activity in order to determine the extent to which I3C is able to activate AhRs in macrophages. The nuclear distribution of AhRs in macrophages in SLE patients and healthy subjects showed an increase of the percentages of positive nuclei compared to the baseline in both SLE and healthy subjects, although the accumulation in the nuclei of the AhR is much greater for SLE patients than for healthy subjects [110].

Despite this, I3C has been introduced as a natural ligand of the AhR with potential therapeutic effects against inflammatory diseases; the mechanism by which the receptor modulates the effects described is not yet clear. AhR-mediated apoptotic activity is essential for the proper functioning of the immune system and for the realignment of aberrant macrophages typical of SLE pathology. The binding between the AhR and a specific ligand with anti-inflammatory properties can also stimulate efferocytosis and decrease the concentration of apoptotic cells in the tissues affected by autoinflammation [114]. I3C is also able to influence estrogen metabolism by diverting it towards the formation of less toxic and more beneficial products. This may be because the AhR can regulate the activity of the ERs, through yet unknown molecular mechanisms. The dialogue between AhR and ER, and the direct effect that I3C has on AhRs, could be the basis for proposing a plausible molecular mechanism [115].

### 2.5. I3C Antiviral Activity and Potential Application on COVID-19 Therapy

I3C (and DIM) antiviral actions are believed to be attributed to estrogenic metabolites deriving in the acidic environment following oral intake [95,116,117] and have demonstrated potent activity against human papillomavirus (HPV) [118]. These derivatives, once formed, bind the cell membranes of the laryngeal tissue and this binding is strongest in the virus tissue [119]. The estrogen treatment has been widely used in the past years but I3C (and DIM) therapy is considered more appropriate since it seems to optimize the balance in estrogen metabolism and reduce the risk of HPV-related pathogenicity [118]. For this reason, with reference to papilloma as a model studied, I3C has been shown to be a potential therapeutic agent against RRP (recurrent respiratory papillomatous) [120].

Viruses cause a wide spectrum of clinical diseases, the majority being acute respiratory infections. In most cases, acute respiratory infection symptoms are similar for different viruses, although the severity can be variable [121]. Viral infections generally trigger a vigorous immune response that is crucial for viral clearance, with a cascade of events involving both the innate and adaptive immune arms in most of cases [122,123].

Respiratory viral infections represent a significant threat to human health worldwide. Influenza is the most common cause of morbidity and mortality both in adults and children with respiratory viral infections. Implementation of molecular diagnostics has demonstrated that other viruses, such as metapneumovirus, are also significant contributors to the overall burden of respiratory infections [124,125]. Besides influenza, COVID-19, an infectious disease caused by SARS-CoV-2, rapidly became a pandemic worldwide. SARS-CoV-2 is a novel coronavirus belonging to the Beta-coronavirus genus. It has a linear, positive-sense single-stranded RNA genome of about 30 kb with an 86% similarity with the SARS-CoV genome [126].

Different approaches are being explored to discover new drug candidates, aimed at identifying molecules able of blocking the entry of the virus into cells, its intracellular replication, and viral exit. For example, several lines of research have demonstrated that SARS-CoV-2 neutralizes antibodies, and synthetic peptides (like DPP4) [127,128] that bind directly to the virus’ spike glycoprotein and inhibit entry into host cells are therapeutically effective. About 374 COVID-19 vaccines have been designed, produced, and are in pre-clinical and clinical development [129,130]. Although vaccines have proven to be very effective against infections and severe symptomatic diseases, the duration of protection of the vaccines available today decreases within 3–6 months [131], as evidenced by the rates of breakthrough infections caused by new variants of the virus [132]. It is, therefore, evident for the need to design and develop new potent drugs able of acting against all variants of the active virus and possibly of eradicating SARS-CoV-2 that have remained incorporated in the patient’s tissues [133]. Numerous drug treatments that target key virus-host interactions, delay the spread of the virus, and reduce the impact of the disease are currently being evaluated in randomized clinical trials [134,135,136]. Despite efforts, specific drugs against COVID-19 have not yet been developed, and this mainly stems from the poor knowledge of host cell drug targets necessary for virus replication and development [137].

Recently, I3C displayed potent anti-SARS-CoV-2 effects. Novelli et al. [10] demonstrated that HECT proteins are involved in SARS-CoV-2 pathology, physically interacting with and ubiquitylating the SARS-CoV-2 spike protein. They showed that some members of the HECT family are expressed in greater quantities in the human cells of infected subjects and in the mouse models of COVID-19. Moreover, they found that several rare variants in the NEDD4 E3 ubiquitin-protein ligase (NEDD4) and WW domain containing E3 ubiquitin-protein ligase 1 (WWP1) genes are associated with severe cases of COVID-19, when compared to asymptomatic controls. In addition, they proved that I3C is able to block SARS-CoV-2 viral egression in the Vero E6 model through the inhibition of HECT proteins implicated in COVID-19 pathology. The importance of E3 ligases in the ubiquitination of some viral proteins has recently been confirmed by combined multi-omics studies that have allowed the demonstration of the ability of SARS-CoV-2 not only in the remodeling of innate immunity, but also in promoting viral infection, by hijacking specific processes of ubiquitination [138]. The role of ubiquitination processes in the survival of the virus have also been very recently confirmed by a study that demonstrated how ubiquitin variants are able to inhibit the production of new viral particles after infection, thus preventing the virus from spreading from one cell to another and wreaking havoc in the body [139]. Furthermore, treatment of human lung organoids (hLORGs) with I3C at different conditions significantly reduces viral entry and the expression of genes involved in innate immunity and inflammation response [11]. Overall, these data suggest the potential use of I3C as an antiviral in clinical trials for patients with COVID-19.

### 2.6. I3C Antimicrobial Activity

Even if most of the current data support the role of I3C (and DIM) in preventing cancers, SLE, and viral infection, it seems that their application in the reduction and prevention of other diseases, such as antimicrobial infections, obesity and diabetes, and cardiovascular diseases, is also possible.

I3C has also proven antibiotic and antifungal activities against human pathogenic microorganisms. Sung et al. have shown that certain Gram-positive bacteria (*Staphylococcus aureus*, *Staphylococcus epidermis*, and *Enterococcus faecium-resistant*), Gram-negative bacteria (*Escherichia coli* and *Pseudomonas aeruginosa*) resistant to drugs, and some species of fungi (*Candida albicans*, *Trichosporon beigelii*, and *Saccharomyces cerevisiae*) are susceptible to I3C [140]. The minimum inhibitory concentration (MIC) of I3C is between 5 and 20 μg/mL for Gram-positive bacteria, 20 to 80 μg/mL for Gram-negative bacteria, and 10 to 20 μg/mL for fungi. This study suggests that I3C exerts its effect by acting on the plasma membrane. A different study has shown that I3C performs a considerable synergistic activity with the antibiotic ampicillin against both Gram-positive and Gram-negative resistant bacteria [141]. The antimicrobial action of I3C is related to its ability to adhere to the phospholipid membrane (in particular lipopolysaccharides) and affect the cytoplasmic membrane and DNA [141]. Unfortunately, the exact mechanism of action remains unclear.

The fungicidal activity of I3C against *C. albicans* is due to its ability to bind to DNA and alter its functions [142]. In addition, dietary supplementation with I3C has been shown to prevent and increase the efficacy of treatments against *Clostridium difficile* in mice [143]. Moreover, it has been shown that food-derived I3C supplementation in mice can prevent and optimize the treatment of an inflammatory process caused by *C. difficile* through mechanisms both dependent and independent of the AhR [143]. Additionally, Sung and Lee (2008a, 2008b), studying the antibacterial activity of I3C, realized that this was inhibited by lipopolysaccharides (LPS) of the wall rather than by the activity of efflux pumps resistant to different drugs (MDRs) located on the outer membrane of Gram-negative bacteria.

Therefore, from many studies, it is clear that the antimicrobial mechanism of action of I3C lies in the deterioration of the plasma membrane, although it is right to emphasize the not-complete knowledge of the mechanism [142].

### 2.7. I3C Antioxidant Activity

Several plants, among which cruciferous vegetables stand out, have proven antioxidant activities [144]. Lipid peroxidation consists of the oxidative degradation of lipids by unsaturated chains and causes damage to cells that can compromise their survival. This process originates mainly from the presence of free radicals, and in vitro studies have shown that I3C limits such damage by increasing cell survival. Such properties have also been confirmed by in vivo studies on mice in which administration of I3C inhibited lipid peroxidation induced by carbon tetrachloride (CCl_4_) with a value of IC_50_ of the order of 35–40 μM [145].

By a molecular analysis, the combination of I3C with crambene (a nitrile product of progoitrin glucosinolate) resulted in a synergistic increase in hepatic quinone reductase mRNA levels in HepG2 cells following the co-activation of antioxidant response elements [146]. It was hypothesized that I3C and crambene, juxtaposed synergistically to each other, are involved in the increased expression of metabolization enzymes.

In contrast, other indole derivatives, such as indole-3-aldehyde and indole-3-carboxylic acid, have no antioxidant activity [147].

### 2.8. I3C Antiobesity Activity and Chronic Diseases

I3C has a potential advantage in the prevention of obesity and metabolic disorders because its action involves multiple mechanisms, including the reduction of adipogenesis and inflammation, and activated thermogenesis. Adipose tissue, in fact, is an endocrine organ associated with states of chronic inflammation and insulin resistance, with the consequence of the inflammation of the adipose tissue itself during normal development and obesity. It also plays an important role in metabolism and is associated with states of chronic inflammation and insulin resistance [148] through the production of cytokines and lipids, with consequences on glucose metabolism, thermogenesis, and adipogenesis [149]. In vitro experiments have shown that I3C reduces lipid particle accumulation in adipocytes and suppresses adipocyte-induced angiogenesis in endothelial cells [150]. These results highlight that I3C represents a potential therapeutic agent for obesity-related diseases, such as type 2 diabetes and atherosclerosis, as demonstrated by a study by Chang et al. in 2011 [151]. All these studies constitute, then, the rationale of the therapeutic potential attributed to I3C in treating obesity and the problems caused by chronic inflammation associated with it.

### 2.9. Other I3C Activities

Besides all of the activities presented in the previous paragraphs, I3C also exerts a neuroprotective effect against neurotoxicity via its anti-inflammatory and antiapoptotic effects. For the first time, Mohamad et al. reveal the promising neuroprotective effects of I3C against rotenone (ROT)-induced Parkinson’s disease in a male albino rat model, exhibiting the highest protection at the dosage of 100 mg/kg/day. These effects were also characterized behaviorally, neurochemically, and histologically, suggesting that I3C neuroprotective effects might be attributed to activating the SIRT1-AMPK signaling pathway that hinders inflammatory and apoptotic cascades [152].

## 3. Natural and Synthetic Derivatives of I3C: (Bio)Synthesis, In Vitro, and In Vivo Activity, and Mechanisms of Action

I3C is metabolically unstable and when orally administered, decomposes under the gastric pH conditions leading to a very rapid acid-catalyzed oligomerization [153,154]. Therefore, one objective has been to make changes to the chemical structure characteristic of I3C in order to improve its stability, but also to study the compounds formed after the decomposition because they are able to produce many of the physiological effects resulting from the administration of I3C [155]. To this goal, some design strategies were applied to improve the possibility of performing lead optimization and obtaining derivatives with a better pharmacodynamic and pharmacokinetic profile than I3C. Thus, the indole ring was functionalized by introducing various substituents with different properties, such as those relating to electronegativity, lipophilicity, and steric hindrance or based on the insertion of electrowithdrawing groups on the nitrogen atom.

Several synthetic strategies reported in the literature to obtain I3C derivatives will be discussed below.

### 3.1. Biosynthesis of I3C Endogenous Derivatives

**Figure 4:**
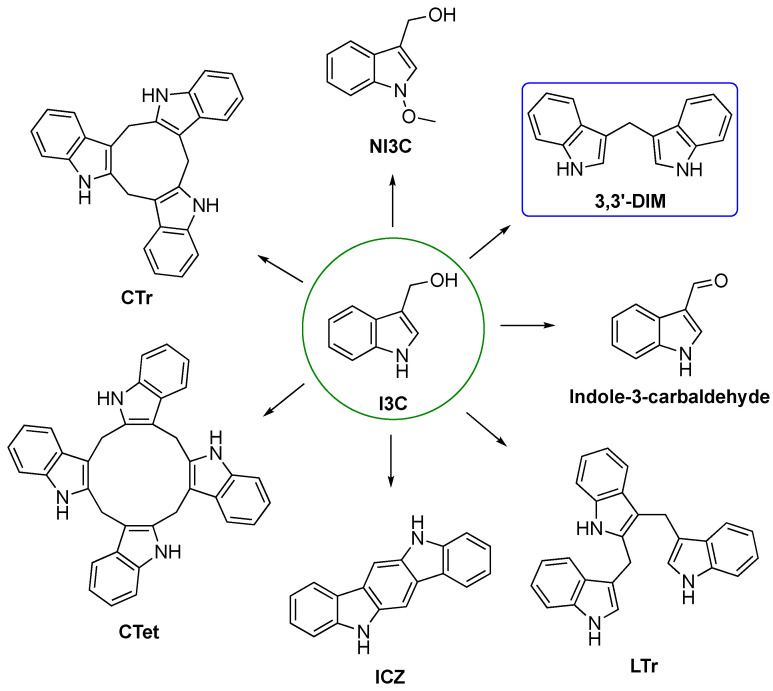
Chemical structures of the main biosynthetic oligomer compounds derived from I3C. 3,3′-DIM is in a box because it is the major and most studied I3C metabolite.

I3C is converted to a series of oligomeric products (Figure 4), which contribute to the in vivo effects of I3C. The main oligomeric compound is 3,3′-diindoylmethane (3,3′-DIM or DIM), which is produced by an acidic environment [7,8]. 5,6,11,12,17,18-hexahydrocyclonona [1,2-*b*:4,5-*b*′:7,8-*b*″]triindole (CTr) is the major trimeric product [156,157,158], whereas 5,6,11,12,17,18,23,24-octahydrocyclododeca [1,2-*b*:4,5-*b*′:7,8-*b*″:10,11-*b′′*′]tetraindole (CTet) is the cyclic tetramer derivative [153,159]. Other derivatives of I3C include indole-3-acetonitrile, indole [3,2-*b*]carbazole (ICZ) [156,160], and indole-3-carboxaldehyde (I3A) [154]. In some cruciferous vegetables, another indole, i.e., *N*-methoxyindole-3-carbinol (NI3C), found in addition to I3C [161], could be potentially used as a pre-treatment in different types of cancer [162]. Another resulting indole oligomer produced in the acidic environment of the stomach is the linear trimer 3,3′-[(1*H*-indole-2,3-diyl)bis(methylene)]bis(1*H*-indole) (LTr) [156,157,163].

**Figure 5:**
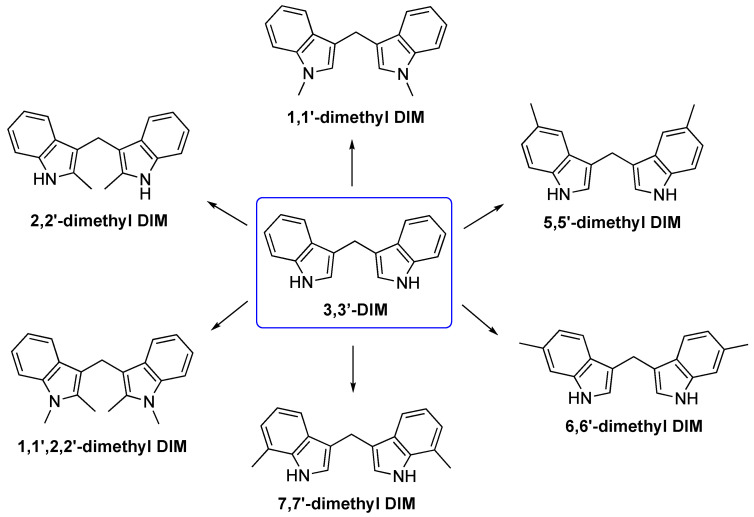
3,3′-DIM derivatives variously substituted.

Other interesting derivatives are DIM substituted with dimethyl groups in different positions, such as 1,1′, 2,2′, 5,5′, 6,6′, 7,7′, or tetramethyl (1,1′,2,2′) [155] (Figure 5).

**Scheme 3:**
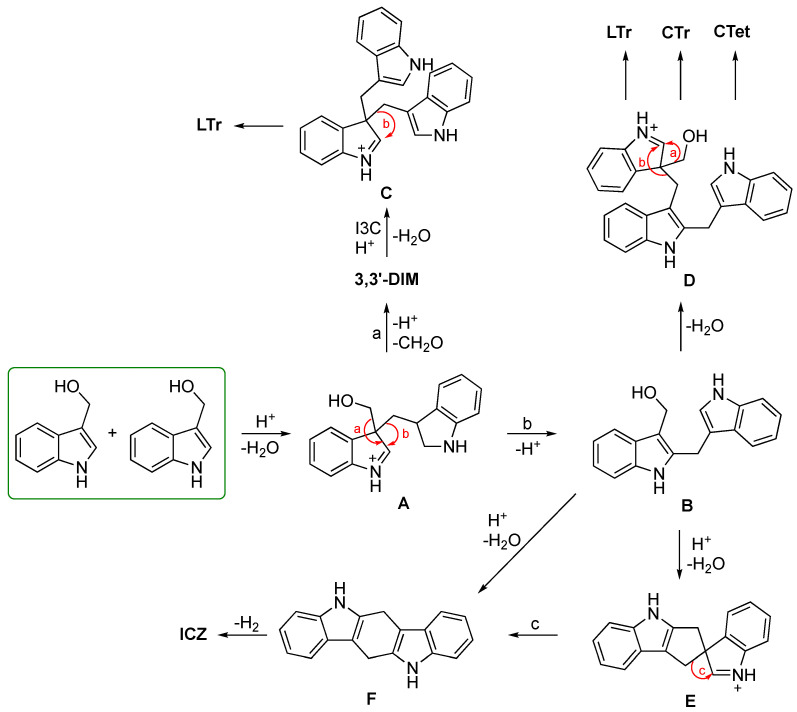
Possible mechanistic details of oligomerization I3C in an acidic environment.

Possible mechanistic details of I3C oligomerization in aqueous acid are provided in Scheme 3 to obtain the main endogenous derivatives. I3C undergoes dehydration in an acidic environment providing 3-methyleneindoleninium cation intermediate **A**, which can either remove formaldehyde (process “a”) to form 3,3′-DIM or is involved in a sigmatropic 1,2-rearrangement (process “b”) leading to compound **B**. The intramolecular attack of **B** directly generates the possible intermediate dihydroindolocarbazole **F**, which can also be produced indirectly by the attack of C-3′ to obtain intermediate **E** via a sigmatropic rearrangement (process “c”). Both processes lead to the formation of ICZ. The intermolecular reaction of **B** with I3C leads to the formation of intermediate **D**, which can lose formaldehyde (process “a”) to give LTr or undergo shift “b”, followed by cyclization to give CTr or react with I3C to produce CTet by similar processes (“d”). However, the electrophilic attack of **A** on 3,3′-DIM produces the intermediate **C**, which can rearrange to form LTr [153].

### 3.2. Synthetic Methodologies of I3C Exogenous Derivatives

The first molecules to be analyzed are the 2,3′-derivatives, which are to be considered, on the one hand, compounds obtained by duplicating I3C and, on the other, molecular simplifications of CTet.

**Scheme 4:**
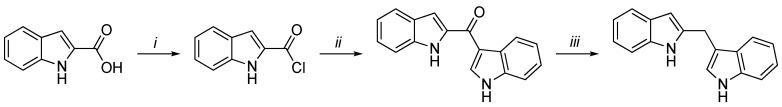
Reagents and conditions: (*i*) SOCl_2_, dry DMF/CH_2_Cl_2_, N_2_, rt, 6 h; (*ii*) indole, Et_2_AlCl, dry CH_2_Cl_2_, N_2_, −15 °C then rt, 4 h; (*iii*) LiAlH_4_, dry THF, N_2_, reflux, 1 h.

The most common procedure to obtain 2,3′-derivatives was proposed by Wahlström et al. through condensation reactions (Scheme 4). The reaction involves the use of diethylaluminium chloride (Et_2_AlCl) to promote the Friedel–Crafts acylation reaction followed by a reduction of the ketone [164].

**Scheme 5:**
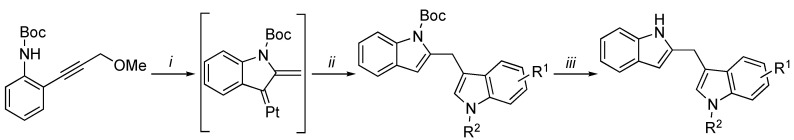
Reagents and conditions: (*i*) PtCl_2_ (5%), Na_2_CO_3_, dioxane, 100 °C; (*ii*) differently substituted indole, 16 h; (*iii*) 160 °C, 2 h.

Winston-McPherson et al. [165] report the synthesis of 2,3′-derivatives from *tert*-butyl [2-(3-methoxyprop-1-yn-1-yl)phenyl] carbamate (propargylic ether) (Scheme 5). Platinum (II) chloride catalyzes the annulation reaction, leading to the formation of the unstable intermediate reported, which reacts with an indole molecule. The last step is to remove the *t*-butyloxycarbonyl (Boc) protective group.

Below ICZ is described, the natural derivative of I3C to be considered a benzofused analog of dimeric derivatives.

**Scheme 6:**
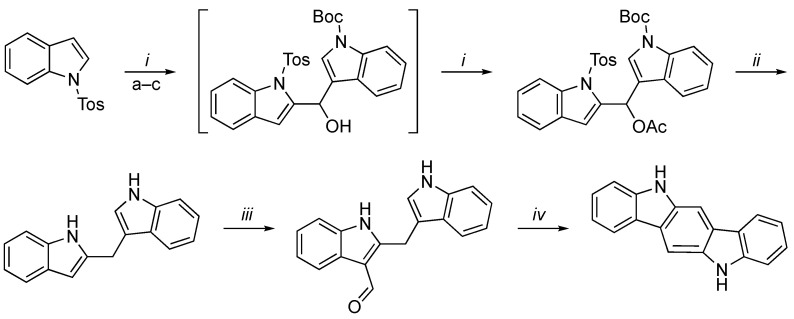
Reagents and conditions: (*i*) (**a**) *t*-BuLi, THF, −20 °C, 45′; (**b**) *N*-Boc-(indole-3-carbaldehyde), THF, 1 h; (**c**) CH_3_COCl, −20 °C to rt, 16 h; (*ii*) Na, liquid NH_3_, THF, −78 °C, 30′; (*iii*) POCl_3_, DMF, 0 °C for 20′ then 40 °C until completion; HCl cat., THF, reflux, 16 h; (*iv*) cat. HCl, THF, reflux.

In the following scheme (Scheme 6), Wille et al. proposed the synthesis of 2,3′-methylenebisindole starting from *N*-tosylindole and *N*-Boc-(indole-3-carbaldehyde) to obtain an unstable intermediate, which reacts immediately with acetyl chloride. The following step is the reaction of the resulting acetate with sodium in liquid ammonia, which led to the reductive removal of the acetoxy group and the simultaneous cleavage of all protective groups [166]. The compound 2,3′-methylenebisindole is used to synthesize intermediate malassezin, which is directly transformed in the final compound.

The importance of CTet is related to the need for in-depth studies regarding this active metabolite of I3C, which differs considerably from the parent compound both for its complexity (a cyclic tetrameric structure) and its very poor solubility in water, in a buffer, and in most organic solvents [167].

**Scheme 7:**
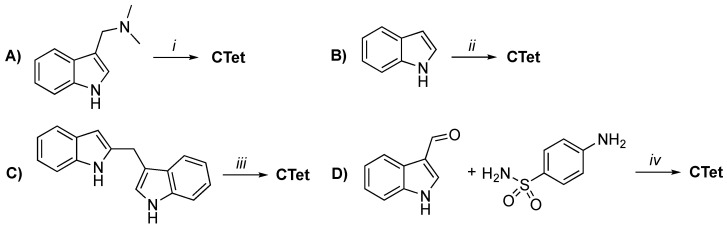
Reagents and conditions: (*i*) (Me)_2_SO_4_, Na, EtOH, rt, 4 h; (*ii*) HC(O)H 37%, MeOH, H_2_SO_4_ 96%, reflux, 2 h; (*iii*) HC(O)H 37%, MeOH, H_2_SO_4_ 96%, reflux, 1 h; (*iv*) AcOH, PtO_2_, H_2_, 60 °C, 4 h.

Four different procedures (**A**–**D**) are reported for the synthesis of CTet (Scheme 7). The methods lead to a mixture of CTr:CTet in a variable ratio from which pure CTet is then obtained. In the first case (**A**), the medium is a preparative reverse-phase HPLC of the crude mixture product [168]. The second (**B**), carried out by a modified synthetic method because it was not reproducible [169], and the others (**C**,**D**) gave CTet after recrystallization of the CTr:CTet mixture from acetone [167,170,171].

**Scheme 8:**
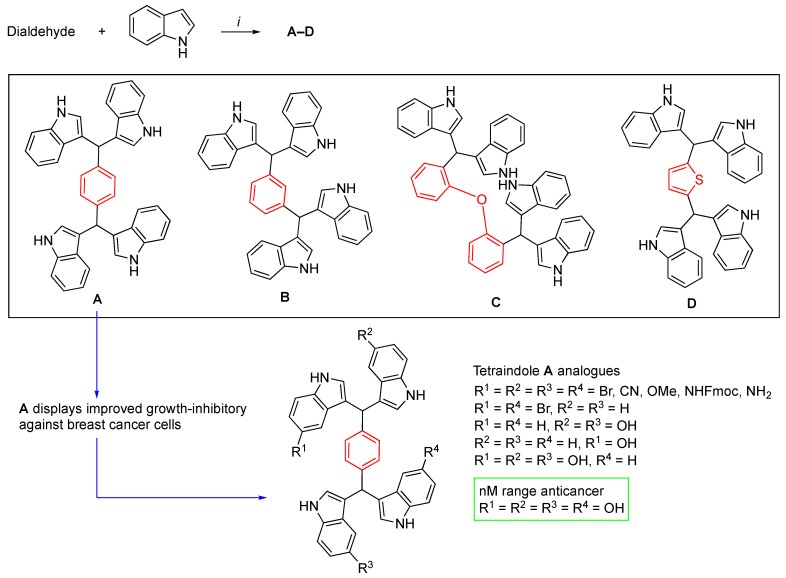
Reagents and conditions: (*i*): I_2_, CH_3_CN, rt, 2 h.

The following chemical structures (**A**–**D**, Scheme 8) represent acyclic tetraindole derivatives, owing a linker with different size and characteristics, able of influencing the spatial orientation of the indole portions and the solubility.

They and their functionalized derivatives are obtained from indole and different dialdehydes (Scheme 8) [172]. The reaction involves the use of molecular iodine. In the case of the most studied derivative (A) an illustrative explanation on its analogues is given in Scheme 8.

**Scheme 9:**
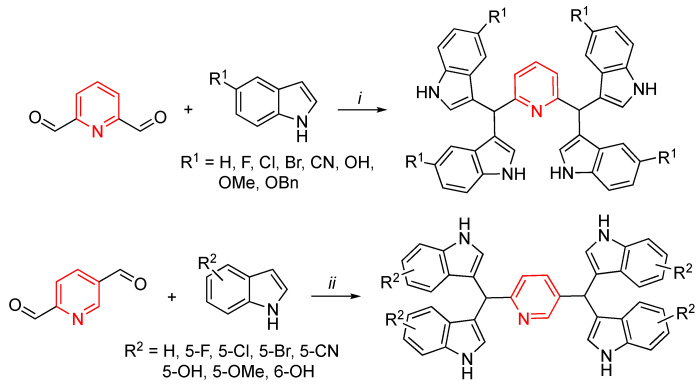
Reagents and conditions: (*i*): CAN, CH_3_CN, reflux, 3 h; (*ii*): I_2_, EtOH, reflux, 6 h.

Tetraindoles possessing 2,6-pyridine and 2,5-pyridine as the core structure have been synthesized [173] (Scheme 9). The syntheses were proposed by Fu et al. by exploiting 2,6-dicarbaldehyde and 2,5-dicarbaldehyde, respectively. A catalytic amount of calcium ammonium nitrate (CAN) was employed for the synthesis of 2,6-derivatives, whereas molecular iodine was used for 2,5-derivatives. The starting indoles used are variously substituted with electrowithdrawing or donating a group on the aromatic core.

Finally, some exemplifying synthetic routes to obtain 3,3′-derivatives are reported below.

**Scheme 10:**
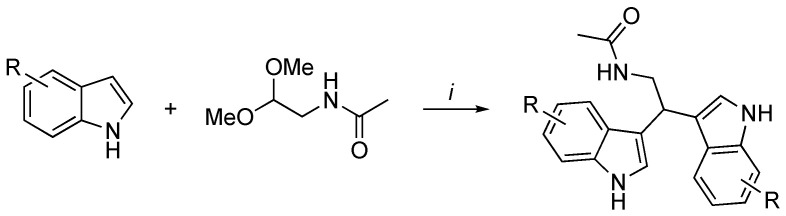
Reagents and conditions: (*i*): DPP (10%), MeCN, 80 °C, 5 h.

Simple and highly efficient methods have been developed due to the possibility of exploiting the good nucleophilicity of the indoles 3-position. One of the common methods to obtain 3,3′-derivatives requires indoles, nitrogen-protected glycin aldehydes, and diphenyl phosphate (DPP) as the organocatalyst, as proposed by Mari et al. [174] (Scheme 10). In detail, indoles with different electrodonating or electrowithdrawing functional groups on the benzene ring as well as alkyl and aryl substituents gave the corresponding bisindoles with good to high yields. In contrast, no reactivity was observed with indoles bearing an electrowithdrawing group at the C-2 position, such as 2-indole-carboxylic acid or its ester, probably due to a combination of steric hindrance and electron deficiency.

**Scheme 11:**
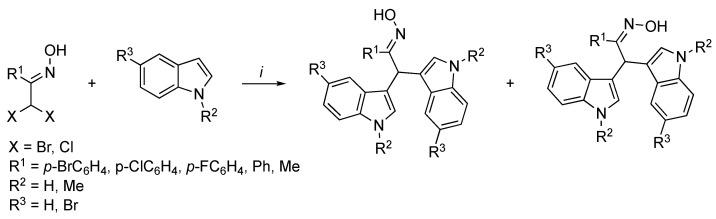
Reagents and conditions: (*i*): Na_2_CO_3_, H_2_O/CH_2_Cl_2_, rt, 36 h.

The group of Grosso et al. [175], started by α,α′-dihalogeno oximes to prepare a variety of new compounds with different substituents at the methylene bridge based on two consecutive hetero Diels–Alder cycloaddition reactions, is shown in Scheme 11.

**Scheme 12:**
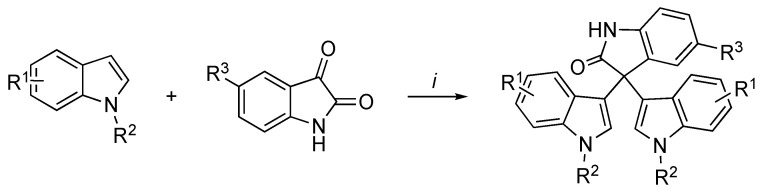
Reagents and conditions: (*i*): FeCl_3_ (5 mol%), MeCN, rt, 15–60′.

Kamal et al. developed an efficient method to synthesize 3,3′-diindolyl oxyindoles employing FeCl_3_ as the catalyst [176] (Scheme 12). The reaction probably involves the activation of the carbonyl group of the isatin moiety and the indole ring by Fe(III). The indoles with electrowithdrawing substituents react more efficiently with isatin and require short reaction times.

**Scheme 13:**
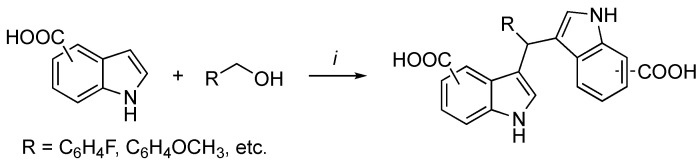
Reagents and conditions: (*i*): Pd(OAc)_2_ (5 mol%), phosphine sulfonate (10 mol%), H_2_O, 60 °C or 80 °C, 16 h.

Hikawa and Yokoyama studied the synthesis of bis(indolyl)methanes by taking advantage of the activation of C-H at position 3 [177]. Indoles with carboxylic acids on the benzene portion were coupled to benzyl alcohols, using Pd(OAc)_2_ in the catalytic amount and a phosphine sulfonate in water (Scheme 13).

**Scheme 14:**
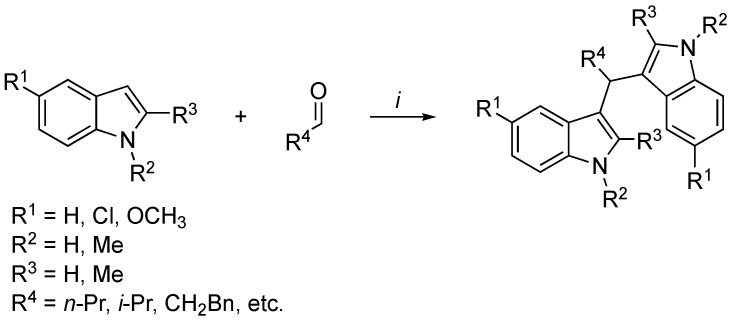
Reagents and conditions: (*i*): AgOTf (1–3 mol%), CHCl_3_, rt, 8–48 h.

The synthesis of 3,3′-derivatives has also been achieved by exploiting silver trifluoromethanesulfonate (AgOTf) as a catalyst from indole and aldehyde, obtaining good yields [178] (Scheme 14).

**Scheme 15:**
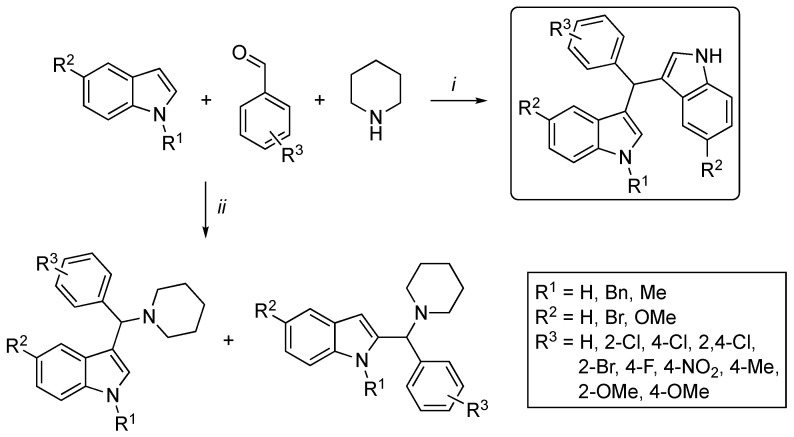
Reagents and conditions: (*i*) solid supported Brønsted acids (CellSA, StarSA) 10 mol%, 1 h, rt; (*ii*) SDS 20 mol%, H_2_O, 80 °C, 2 h.

Kumar et al. [179] reported a highly efficient procedure for the synthesis of 3,3′-DIM via a multicomponent Mannich-type reaction between indole, aromatic aldehyde, and secondary amine providing 3-amino alkylated indoles (Scheme 15). In water and sodium dodecyl sulfate (SDS), 3-amino alkylated indole derivatives were effectively obtained, while in the presence of an organic solvent and a solid support Brønsted acid, the bisindolic compound was obtained with high selectivity.

**Scheme 16:**
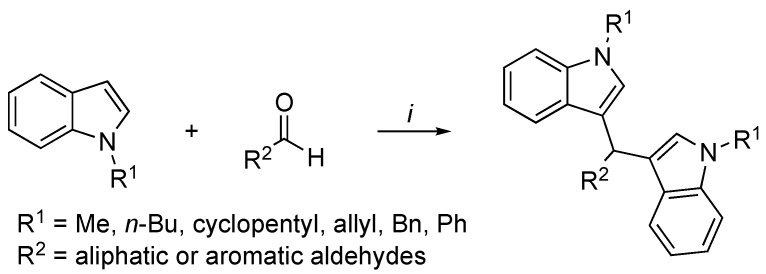
Reagents and conditions: (*i*) arylazo sulfone derivative (0.5 mol%), hν (460 nm), CHCl_3_, rt, 6 h.

The Friedel–Crafts reaction is the most common method to functionalize aromatic rings, including indoles. An economical and sustainable method to obtain 3,3′-derivatives by exploiting the photochemical arylation of aldehydes has been developed [180] (Scheme 16). This method is based on the use of an arylazo sulfone derivative for the visible-light photochemical catalytic release of acid under blue LED lamp irradiation. This slow-release acid activates aromatic or aliphatic aldehydes. *N*-(cyclo)alkyl, *N*-allyl, *N*-aryl, or *N*-arylalkyl substituents, such as those indicated in the scheme, are well tolerated and lead to the desired products in good yields.

### 3.3. In Vitro and In Vivo Activity and Mechanism of Action of I3C Derivatives

The most bioactive constituent derived from I3C is 3,3′-DIM (DIM) derivatives; many mechanisms of action have been described for these compounds, especially regarding to the modulation of carcinogenesis, as previously mentioned from a comparison of I3C and DIM. Several studies conducted with dimeric products suggest that they are able to deregulate multiple cells’ signaling pathways that are essential for tumor growth and spread [181].

This has been seen in multiple studies influencing ER signaling in the MCF-7 breast tumor line [23,44,74,75,76,78,79,81,158,182,183]. A type of tumor in which attention has been paid, and which has led to consider the organ target of chemoprevention operated through DIM, is that of the prostate, due to the antagonism of DIM on the androgen receptor [44]. Other mechanisms are related to the induction of apoptosis, blockade of NF-κB, Akt, and Wnt, and effects on PI3K/Akt/mTOR signaling [184]. DIM, unlike competitive inhibitors of enzyme activity, causes selective proteasome degradation [185,186] of HDAC class 1 enzymes (HDAC 1, 2, 3, 8) in human colon and prostate cancer cell lines; this new mechanism could be exploited in the clinic with HDAC inhibitors to achieve lower histone acetylation and/or a lower concentration of the necessary HDAC inhibitors. In some mouse colon cells and human Caco-2 cells, HDAC inhibitors led to increased ligand-dependent induction of *CYP1A1*, AhRs, and other AhR target genes [187] including the *CYP450 1B1* gene, which hydroxylates estrogen at positions 2 and 4 [188]. DIM seems to have an inherent action both with the AhR-dependent up-regulation of enzymes, metabolizing drugs and carcinogens, such as CYPs, GSTs, UGTs, and SULTs, and with the in vitro inhibition of the catalytic activity of cytochromes [154]. DIM has also been shown to prevent liver cancer formation and protect liver tissue through suppression of TGF-β, Smad2/Smad3, AP-1, and NADPH [189].

Antiproliferative effects in vitro and in animal models are reported in the antibiofilm activity of DIM and its derivatives against common gram-negative bacteria of a medical purpose. However, the effect of DIM on biofilm formation, extracellular matrix closely related to biofilm of gram-positive foodborne bacteria *S. aureus*, and its potential application prospect in the food industry are still unknown [190].

DIM could be a potential therapeutic agent to treat obesity and obesity-associated disorders because it reduces body weight and fat in animals fed a high-fat diet by inhibiting preadipocyte differentiation. Due to the fact that AhR receptors are a crucial transcription factor in adipogenesis and angiogenesis, DIM most likely carries out its antiobesity activity via these receptors [189] and, thus, decreases the development of several chronic diseases (including diabetes) such as cardiovascular disease and cancer.

DIM and derivatives help to control the proliferation and abnormal differentiation of Vascular smooth muscle cell (VSMC), in which proliferation is associated with several pathological conditions, including restenosis, atherosclerosis, and hypertension. In these cases, phenotypic modulation of VSMCs results in loss of contractility, matrix secretion, migration, and abnormal proliferation [191] by regulating several signaling pathways such as mitogen-activated protein kinase (MAPK) and AKT/PI3K, and by inhibiting platelet-derived growth factor (PDGF) induced proliferation of VSMCs and causing cell cycle arrest in a dose-dependent manner [63].

Similary to I3C, its DIM metabolite also shows antioxidant [192] and radical scavenger activity, sometimes even higher. In this scenario, DIM has been shown to be more effective as an antioxidant agent than I3C, compared to metformin, which is considered the standard reference drug [193].

Formulated DIM (BR-DIM or B-DIM = BioResponse-DIM) has been well tolerated by healthy subjects and it is used in clinical trials for the treatment of breast cancer and prostate cancer [184]. Specifically, BR-DIM appears to be able to inhibit prostate tumor growth via upregulation of suppressor microRNAs (miRNAs) [194].

On the other hand, in vivo, supplementation with DIM required great attention in order not to incur an adverse “drug–drug” interaction that, according to some results deriving from clinical studies conducted on sick subjects and with the exclusion of criteria that minimize the potential risks of such adverse interactions, does not seem to manifest itself with a long-term supplementation of 300 mg/day. A further possible risk and opportunity is characterized by the mutual interaction between the DIM and the human microbiome of the individual; in fact, a DIM-AhR-intestinal microbiome axis has been unraveled, which could represent an important factor for the design of a “personalized nutraceutical” approach to achieve significant benefits [154].

With regard to cyclic tetrameric derivatives CTr and CTet, it was demonstrated that both are able to exert an anticancer activity.

CTr increases proliferation of estrogen-dependent breast tumor cells, binds with a strong affinity for the estrogen receptor-alpha (ERa), and activates the expression of the estrogen (E2)-dependent gene [158,195].

In a study performed by De Santi et al., the breast cancer cell lines MCF-7 (ER positive) and MDA-MB-231 (triple-negative) have been analyzed in response to CTet treatment in terms of cell cycle perturbations and autophagy induction [170], and the ER stress response was considered as the main upstream CTet molecular mechanism for both MCF-7 and MDA-MB-231 cells [196]. The identification of the overexpression of p21/CDKN1A (Cyclin-Dependent Kinase inhibitor 1A) due to the inhibition of Akt activity as the strongest molecular event induced by CTet treatment in MCF-7 and p53-mutant MDA-MB-231 cells was obtained [170]. Another study demonstrated the synergistic activity of CTet in combination with cisplatin and doxorubicin in breast cancer cells in terms of cell viability, induction of autophagy, and overexpression of MAP1LC3B (microtubule-associated proteins 1A/1B light chain 3B) [197]. Finally, an interesting study concerning the CTet inhibition of testosterone aromatization in CYP19A1-overexpressing MCF-7 breast cancer cells is to be included in the present description [198].

Moreover, a mixture of tri- and tetrameric cyclic I3C derivates formed by CTr/CTet has been tested in vitro and in vivo. In vitro, this mixture was able to inhibit breast cancer cell proliferation, inducing the G0/G1 cell cycle phase accumulation. From a molecular point of view, CTr/CTet mixture is able to: overexpress p21, p27, and GADD45A (growth arrest and DNA damage inducible alpha), promote nuclear translocation of FOXO3a, inhibit Akt activity, and downregulate estrogen receptors. Similarly, the CTr/CTet mixture inhibits the growth of xenotransplanted tumors through the downregulation of cyclin E. For the first time, this data also highlights the potential anticancer activity of the I3C mixture in the treatment of triple-negative breast and hormone-responsive cancer [16].

The synthetic pyridine-based tetraindole (PBT) derivatives were observed to display the highest antiproliferative activity against the two triple negative breast cancer (TNBCs) cell lines. Its mechanism of action was shown to involve G2/M arrest of the cell cycle [173].

(1*H*-Indole-3-yl)methanol is a breakdown product from cruciferous vegetables. It is included in the family of 2,3′-DIM derivatives and its dimerization product ICZ is a strong agonist of the AhR [166]. The natural product malassezin and ICZ are AhR agonists with an interesting 2,3′-DIM skeleton.

## 4. Conclusions and Perspectives

I3C is a secondary metabolite produced by *Brassicaceae* that, together with its major metabolite DIM and others, has important biological properties. On many of these compounds, there are several studies that encourage biomedical applications, especially the prevention and treatment of inflammatory origin diseases. I3C exhibits low levels of toxicity and performs important activities in multiple molecular and biological processes, including lymphocyte differentiation and carcinogen metabolism. In addition, it has properties related to DNA repair and apoptosis induction in aberrant cells, a situation that places this compound among the most promising future therapeutic agents. Several studies, both experimental and observational, attribute antitumor, anti-inflammatory, antimicrobial, and antiviral properties to I3C and DIM. Other studies hypothesized their activity on different pathologies, also in combination with other drugs, with which it often has synergistic effects, and that try to clarify its molecular biological effects. More recently, I3C has also been evaluated as an antiviral agent for SARS-CoV-2 infection. Studies in this field are preliminary, but they are still extremely promising. The effect of I3C would be to hinder the interaction of the spike protein of the virus with the HECT E3 cell ligases, which through the ubiquitination of target proteins, represents an essential step in the release of virions into infected cells.

Studies and research are still ongoing, and the hope that I3C or its metabolites and derivatives may in the future be the basis for the development of new drugs characterized by high efficacy and selectivity, and accompanied by mild or no side effects, is increasingly concrete. In particular, the most studied derivatives to date are the 3,3′-DIMs. Tetraindoles also show interesting in vitro activity, but the problem is the poor pharmacokinetic profile that leads to difficult administration. Finally, 2,3′-DIM derivatives, whereas there are currently few studied and actually identified as an AhR receptor agonism, are very promising for future studies and applications.

## Data Availability

Data is contained within article.
